# Quantifying Time to Diagnosis of CKD in the United States: Analysis of Electronic Health Records-Linked Retrospective Claims Data

**DOI:** 10.34067/KID.0000001056

**Published:** 2025-12-05

**Authors:** Adrian R. Levy, Satabdi Chatterjee, Sydnie Stackland, Bonnie M.K. Donato, Ling Zhang, Csaba P. Kovesdy

**Affiliations:** 1 Department of Community Health and Epidemiology, Dalhousie University, Halifax, Nova Scotia, Canada; 2 Boehringer Ingelheim Pharmaceuticals Inc., Ridgefield, Connecticut; 3 Panalgo, Boston, Massachusetts; 4 University of Tennessee Health Science Center, Nashville, Tennessee

**Keywords:** CKD, clinical epidemiology, health policy

## Abstract

**Key Points:**

- Many people with CKD are unaware of the condition.
- We estimated the time to CKD documentation after two eGFR measurements taken at least 90 days apart.
- Among many persons with early stage CKD, there are considerable delays in documenting the diagnosis in electronic health record-linked claims data.

**Background:**

An estimated 14% of US adults have CKD with over 90% unaware of their condition. Health care professionals diagnose CKD after two abnormal laboratory test results taken at least 3 months apart. Although there is evidence that delays from CKD onset to documentation are common, these delays remain unquantified. The objective of this study was to quantify the time from laboratory-based evidence of CKD to the documentation of CKD using International Classification of Diseases codes.

**Methods:**

A retrospective longitudinal cohort study was conducted using 2009–2020 Optum Market Clarity data. Adults aged 18 years and older were followed from the date of the second of two eGFRs <60 ml/min per 1.73 m^2^, 3–12 months apart until the first International Classification of Diseases Ninth or Tenth revision diagnosis of CKD, or censoring. Survival analysis was used to compare time to documentation among Kidney Disease Improving Global Outcomes (KDIGO) categories considering while analyzing deaths as competing risks.

**Results:**

A total of 1.39 million adults with laboratory evidence of CKD and a mean age of 71 years (SD, 10; 63% women; 87% White) were included. Over 94% were in KDIGO stage G3, 5% in stage G4, and 1% in stage G5. The median time to CKD documentation was 3.6 years (interquartile range, 1.0–8.4), ranging from 4.8 years for those in KDIGO G3a, 2 years in KDIGO G3b, and <1 year in KDIGO G4 and G5. Patient characteristics associated with longer time to CKD diagnosis included absence of diabetes or heart failure, less severe CKD, older age, and female sex.

**Conclusions:**

There was a substantial delay between laboratory evidence of CKD and the diagnosis being documented *via* coding. Reducing this delay offers a target for earlier recognition and management of CKD.

## Visual Abstract

**Figure:**
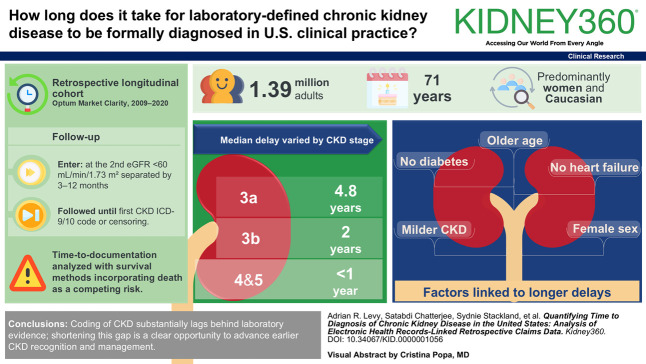

## Introduction

An estimated 14%^1^ of adults in the United States suffer from CKD with a large majority (90%)^2^ unaware of their condition. Declining kidney function leads to complex changes (*e.g*., anemia, altered mineral homeostasis, salt and water retention, inflammation, uremic toxins, hypertension), which can lead to cardiac complications.^3,4^ As a result, patients with CKD are at an increased risk of hospitalizations, cardiovascular events, and all-cause mortality.^5,6^ Early detection and management reduces risk of associated morbidity,^7^ and there are pharmacologic therapies available to slow the decline in kidney function.^8,9^ Patients diagnosed with more impaired kidney function benefit from referral to a nephrologist.^10^

Early detection of CKD, along with risk stratification and treatment, is now recommended for patients at a high risk of the condition.^11^ CKD is diagnosed on the basis of laboratory testing: markers of both kidney function or kidney damage–eGFR values <60 ml/min per 1.73 m^2^ (on the basis of serum creatinine) or albuminuria >30 mg/g—which must be present for at least 3 months.^12^ Diagnosis requires a health care provider to note biochemical abnormalities sustained over time to rule out AKI.^13^ Testing eGFR and urine albumin-creatinine ratio (uACR) may be ordered by automated requests or by health care providers.^14^ In the United States, uACR testing among persons with undiagnosed CKD is very low—at most 3%^11,15–17^—meaning that, in practice, eGFR is the most commonly used tool for detection. Although previous investigators have found rates of diagnosis among patients with CKD documented in their medical records,^18–23^ there is as yet little information on the temporal distribution of CKD documentation.

Documentation of a CKD diagnosis by a health care professional indicates recognition that the patient has the condition and presents an opportunity to align management with evidence-based guidelines. The primary objective here was to quantify the time from laboratory-based evidence of CKD until the first International Classification of Diseases Ninth or Tenth revision (ICD 9/10) code for CKD recorded in electronic health record (EHR)-linked claims data and identify the factors associated with time to documentation.

## Methods

We performed a retrospective cohort study to estimate the time between a laboratory detection and clinical documented diagnosis of CKD. We operationalized the sequence and timing of events in an EHR-linked claims database, a primary source of information for studying the sequence and timing of events in management of chronic diseases.^24–26^ The target population was US adults aged 18 years and older with laboratory evidence of CKD defined by two eGFRs <60 ml/min per 1.73 m^2^, 3–12 months apart.

**Figure 1:**
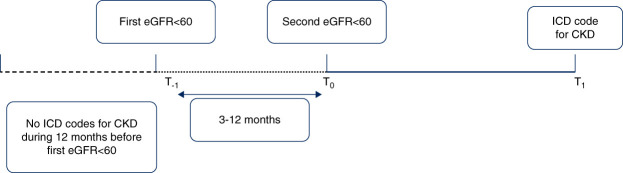
**Temporal sequence for documenting a diagnosis of CKD in EHR-linked claims data.** EHR, electronic health record; ICD, International Classification of Diseases.

The outcome was the time interval measured (in years) between second eGFR <60 ml/min per 1.73 m^2^ and first date of a documented ICD 9/10 diagnosis of CKD (Figure 1).

### Data Source

We used deidentified Optum Market Clarity data that comprises EHR linked to health insurance claims data.^27^ Optum Market Clarity includes data on more than 102 million patients from integrated delivery networks and ambulatory care centers treated by 150,000 medical providers in over 2000 hospitals and 7000 clinics from all regions of the United States^28^ The data are longitudinal in nature and capture detailed information about a patient's history including testing of 400 laboratory tests (using Logical Observation Identifiers Names and Codes^29^). The eGFR was estimated using the CKD Epidemiology Collaboration.^30^ Albuminuria was reported infrequently (<1% of patients) and therefore was not considered for analysis. Diagnoses were coded using the ICD 9/10.

As only deidentified, Health Insurance Portability and Accountability Act compliant data were used, informed consent was not needed and Institutional Review Board approval was not required.

Data were curated and analyzed through a third-party vendor using the Instant Health Data software (Panalgo, Boston, MA; https://panalgo.com/) and R, version 3.2.1 (R Foundation for Statistical Computing, Vienna, Austria).

### Design

The study sample included patients with laboratory evidence of CKD between January 1, 2009, and January 1, 2019. We excluded patients who had evidence of CKD before 2009. Patients were followed for a minimum of 365 days until electronic documentation of CKD with an International Classification of Diseases (ICD) code, death, date of last encounter, or end of follow-up (December 31, 2020). The first day of follow-up–the index date—was defined as date of confirmed laboratory evidence of CKD, *i.e*., a second eGFR <60 ml/min per 1.73 m^2^ measured a minimum of 3 months apart.^15,16^ To operationalize this criterion, we set a maximum 12 months for the second eGFR. A lookback period of 12 months from the index date (second eGFR) was used to assess baseline demographic factors and the Elixhauser Comorbidity Score.^31^ The use of uACR testing was infrequent, with over 97% of patients never being tested, meaning that micro- and macroalbuminuria were not available for detection and risk stratification.

### Analyses

We estimated the cumulative incidence of diagnosis as a function of days since index with death as a competing event. We treated date of last encounter as right-censored observations.^32^ We plotted cumulative incidence functions for each of the Kidney Disease Improving Global Outcomes (KDIGO) categories. The effects of covariates on time to documentation of CKD were estimated by hazard ratios (HRs) along with 95% confidence intervals derived using the Fine and Gray estimator that corrects for the competing risk of death. The effect size for each predictor was estimated while adjusting for the competing risk of death.^33^

## Results

After exclusions (Supplemental Figure 1), there were 1.39 million adults with laboratory evidence of CKD in Optum Market Clarity between 2009 and 2019 (Table 1). The mean age was 71.4 (SD=10; median 74 [interquartile range (IQR), 65–79]) years, and 63% were women. Over half of the patients resided in the Midwest (54%), followed by the South (22%), the Northeast (12%), and the West (9%). At entry, over one half of patients were insured by Medicare Advantage, one-third had commercial insurance, and <5% were insured by Medicaid. At cohort entry, over 90% of patients were in stage G3a or G3b, 5% in stage G4, and about 1% in stage G5. The median (IQR) Elixhauser score was 3 (1–4).

**Table 1:** Demographic and clinical characteristics of patients on the date of laboratory-based evidence of CKD according to Kidney Disease Improving Global Outcomes stage, Optum Market Clarity 2009–2020

Characteristic	KDIGO G3a (*n*=967,677)	KDIGO G3b (*n*=334,866)	KDIGO G4 (*n*=74,893)	KDIGO G5 (*n*=16,342)	All Patients (*n*=1,393,778)
**Demographic characteristics**					
Age (yr.)					
*Mean (SD)*	71 (9.9)	72.8 (9.6)	72.1 (10.8)	63.2 (14.6)	71.4 (10)
*Median (IQR)*	73 (65–79)	76 (68–80)	77 (66–80)	65 (54–76)	74 (65–79)
*Min–Max*	18–86	18–86	18–86	18–86	18–86
Sex					
*Women*	596,842 (61.7%)	219,612 (65.6%)	48,939 (65.3%)	9066 (55.5%)	874,459 (62.7%)
*Men*	370,835 (38.3%)	115,254 (34.4%)	25,954 (34.7%)	7276 (44.5%)	519,319 (37.3%)
Race					
*Asian*	9393 (1.0%)	3249 (1.0%)	797 (1.1%)	230 (1.4%)	13,669 (1.0%)
*Black*	61,336 (6.3%)	24,787 (7.4%)	7547 (10.1%)	3598 (22.0%)	97,268 (7.0%)
*White*	853,333 (88.2%)	289,034 (86.3%)	61,396 (82.0%)	10,960 (67.1%)	1,241,723 (87.0%)
*Missing*	43,615 (4.5%)	17,796 (5.3%)	5153 (6.9%)	1554 (9.5%)	68,118 (5.0%)
**Clinical characteristics**					
Elixhauser score					
*Mean (SD)*	3.2 (2.5)	3.1 (2.6)	3.0 (2.8)	2.3 (2.7)	3.1 (2.6)
*Median (IQR)*	3.0 (1–4)	3.0 (1–5)	2.0 (1–4)	1.0 (0–4)	3.0 (1–4)
Preexisting conditions					
*No HF/diabetes*	234,077 (24.2%)	80,025 (23.9%)	16,539 (22.1%)	3285 (20.1%)	333,926 (24.0%)
*Diabetes alone*	48,705 (5.0%)	21,907 (6.5%)	5879 (7.8%)	749 (4.6%)	77,240 (5.5%)
*HF alone*	74,321 (7.7%)	31,642 (9.4%)	8099 (10.8%)	983 (6.0%)	115,045 (8.3%)
*HF and diabetes*	610,574 (63.1%)	201,292 (60.1%)	44,376 (59.3%)	11,325 (69.3%)	867,567 (62.2%)

HF, heart failure; IQR, interquartile range; KDIGO, Kidney Disease Improving Global Outcomes; Min–Max, minimum and maximum.

Patients were followed for a mean of 2.7 years (SD, 2.4) after laboratory evidence of CKD. Approximately 8% of patients died before a claim for CKD.

Table 2 shows the number of days elapsing for a specified proportion of patients to the first ICD code for CKD across KDIGO stage. Overall, 53.1% of patients had CKD documented by a health care provider during follow-up, ranging from 46.5% in stage G3a, 66.3% in stage G3b, 76.2% in stage G4 and 69.6% in stage G5. For the overall cohort, the median time to CKD documentation was 3.6 years (IQR, 1.0–8.4), ranging from 4.8 years for those in KDIGO G3a, 2 years in KDIGO G3b and <1 year in KDIGO stages G4 and G5. Among those who had a diagnosis of CKD during follow-up, the respective median times to documentation was 1.3 years (IQR, 0.3–3.0) and ranged from 1.7 years in stage G3a, 1 year in stage G3b, and <1 year in stages G4 and G5.

**Table 2:** Distribution of time (days) from the date of laboratory-based evidence of CKD to documentation of International Classification of Diseases diagnosis, according to Kidney Disease Improving Global Outcomes stage

KDIGO Stage	*No.* with Laboratory Evidence of Disease	% with ICD Documentation	Median (IQR)	Tenth Percentile	90th Percentile
G3a	967,6770	46.5	1761 (628–3550)	158	3931
G3b	334,866	66.3	726 (186–1867)	13	3473
G4	74,893	76.2	266 (28–859)	5	2010
G5	16,342	69.6	200 (12–1078)	5	3922
All stages	1,393,778	53.1	1331 (390–3073)	55	3925

ICD, International Classification of Diseases; IQR, interquartile range; KDIGO, Kidney Disease Improving Global Outcomes.

**Figure 2:**
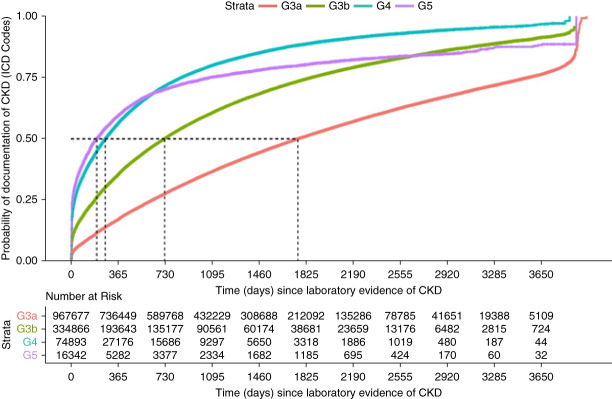
**Probability of ICD documentation of CKD diagnosis within a certain time of laboratory testing indicating CKD, by KDIGO stage, Optum Market Clarity 2009–2020.** KDIGO, Kidney Disease Improving Global Outcomes.

Figure 2 shows the cumulative probability of CKD documentation by time since cohort entry; the steeper the slope of the line, the shorter the time to documentation. The figure shows that the time to documentation was approximately the same for patients in KDIGO stages G4 and G5 for the first 2 years and then was faster for stage G4. The figure also shows that the time to documentation was substantially slower in KDIGO G3b (median 2.0 years) and even slower in G3a (median 4.8 years).

For patients in KDIGO stages G4 and G5, almost 25% were diagnosed within 1 month of laboratory evidence of CKD and then the rates of CKD documentation remained approximately parallel across KDIGO groups. Patients in KDIGO 3b showed a similar pattern although with a substantially smaller proportion diagnosed within the first month (approximately 10%) and those in KDIGO 3a show an even smaller (<5%) at the beginning of follow-up. This was reflected in the first decile which was 0.1 years for G4 and G5, 0.04 years in G3b and 0.43 years in G3a.

Table 3 shows the association between demographic and clinical characteristics and the likelihood of ICD documentation, with HRs above 1.0 indicating a higher likelihood of, and shorter time to, CKD documentation. Men were 50% more likely to have a documented diagnosis of CKD than women (HR, 1.46; 95% confidence interval, 1.43 to 1.49). Other factors associated with a statistically significantly shorter time to documentation included presence of cardiometabolic conditions (diabetes and/or heart failure), non-White race, cohort entry in recent years, and more severe CKD stage. Relative to KDIGO stage G3a, the likelihood of CKD diagnosis was 1.9 times higher in G3b, three times higher in G4, and 2.5 times higher in G5.

**Table 3:** Association between demographic and clinical characteristics and the likelihood of International Classification of Diseases documentation, with hazard ratios >1.0 indicating shorter time to documentation, among 1,393,778 patients with laboratory evidence of CKD in the United States, 2009–2020

Characteristic	HR (95% CI)
**Age group, yr.**	
<50	Referent (1.0)
50–59	0.96 (0.90 to 1.03)
60–69	0.95 (0.89 to 1.02)
70–79	0.92 (0.87 to 0.98)
≥80	0.89 (0.83 to 0.95)
**Sex**	
Women	Referent (1.0)
Men	1.46 (1.43 to 1.49)
**Race**	
Asian	1.18 (1.07 to 1.30)
Black	1.62 (1.57 to 1.68)
White	Referent (1.0)
**Region**	
Northeast	Referent (1.0)
Midwest	1.09 (1.06 to 1.12)
South	0.95 (0.92 to 0.99)
West	1.24 (1.19 to 1.29)
Missing	1.11 (1.04 to 1.18)
**Insurance**	
Medicare advantage	Referent (1.0)
Commercial	0.95 (0.93 to 0.97)
Medicaid	1.06 (1.00 to 1.11)
Missing	1.00 (0.97 to 1.03)
Uninsured	0.95 (0.87 to 1.02)
**Year of cohort entry**	
2010–2011	Referent (1.0)
2012–2013	1.10 (1.07 to 1.14)
2014–2015	1.06 (1.03 to 1.09)
2016–2017	1.11 (1.08 to 1.15)
2018–2019	1.17 (1.12 to 1.22)
**KDIGO stage at baseline**	
G3a	Referent (1.0)
G3b	1.91 (1.87 to 1.95)
G4	2.96 (2.85 to 3.08)
G5	2.49 (2.27 to 2.74)
**Comorbid conditions at baseline**	
No diabetes/no HF	Referent (1.0)
Diabetes	1.32 (1.29 to 1.36)
HF	1.17 (1.12 to 1.21)
Both diabetes and HF	1.60 (1.53 to 1.68)
Mean Elixhauser Comorbidity Score	0.99 (0.98 to 0.99)

CI, confidence interval; HF, heart failure; HR, hazard ratio; KDIGO, Kidney Disease Improving Global Outcomes.

## Discussion

A fundamental challenge impeding widespread treatment of early CKD is that it remains underdiagnosed.^34^ By implementing a simplifying assumption that CKD can be documented after two eGFR measurements, this study presents the entire distribution of time to CKD documentation in a large cohort of insured US adults: only 53% of patients had ICD documentation after a mean of 2.7 years after laboratory evidence of CKD. This finding helps quantify the gaps in early identification identified by KDIGO.^12,34^ With recently completed randomized trials demonstrating that new CKD treatments can slow CKD progression and delay cardiac morbidity,^35,36^ the need for timely diagnosis is critical for management. Quantifying the time to diagnosis following completion of laboratory testing is a key step in characterizing the diagnostic process.^37^ In this large cohort of insured US adults, we observed substantial delays in documentation for those with KDIGO stage G3a, with the median time to an ICD code of almost 5 years. Although the results are consistent with the finding that advanced stages of CKD are more readily recognized and documented by health care providers than milder CKD stages,^38^ we observed that for over one quarter of patients in KDIGO stages G4 and G5, several years elapsed before an ICD code appeared in their records.

The findings are of primary use to payers and other health systems policy makers. By quantifying the delay to diagnosis after laboratory confirmation of CKD, the results provide a baseline to which initiatives to improve the diagnosis of CKD can be compared. The study findings are most applicable to commercially insured adults in the United States who have claims information matched with EHR data. This information provides baseline values against which new policies seeking to accelerate the diagnostic process may be measured.^39^ In addition to the health benefits of early identification and management of CKD, there are substantial financial implications in terms of costs of potential cardiovascular events averted.^40^

There are important limitations to keep in mind when interpreting the results. First, by design, we reduced a multistep pathway for diagnosing CKD in primary care^12^ to two measurements using a lone physiologic measure (eGFR). This means that the delay to documentation developed in this study, developed from a commercial EHR-linked claims database, is a useful proxy for the underlying temporal sequence of clinical and administrative processes required for a diagnosis. Second, we used ICD codes listed on administrative claims to identify the date CKD was documented by a health care professional. As these data source are not collected for research purposes, misclassification is a potential bias. In a review of validation studies of administrative diagnostic codes for CKD described in 19 studies that included 462,563 patients, the findings were as follows: specificity and negative predictive values were high, with medians of 98% and 84%, respectively; sensitivity and positive predictive values were more variable with a median sensitivity of 41% (range 3%–88%) and a median positive predictive value of 78% (range 29%–100%).^41^ These data can be interpreted to mean that if an ICD code for CKD was present, we can be more confident the patient had the condition. Third, the date of the first ICD diagnosis for CKD was used as a proxy for the date of diagnosis. The assumption is that after ordering testing, the clinician can start managing CKD.^42^ Fourth, we minimized selection bias that occurred during follow-up by censoring patients on their date of last encounter with the health care system. The distributions of race and geographic region reflected the data included in Optum Market Clarity and differed from the population of the United States, with overrepresentation of White patients and those from the Midwest and underrepresentation of Black and Asian patients and from other regions in the United States (data not shown). The estimated time to documentation of CKD likely underestimates that parameter among those without health insurance. Even if the results on the basis of an EHR claims-linked database do not reflect the entire US population, any future changes in time to diagnosis would likely be reflected in the data. This means that these results could be used as baseline for a future study with Optum data. Fifth, like other US studies,^11,15–17^ the use of uACR testing was very low in this cohort (over 97% of patients without the test). This precluded its use in identifying and risk-stratifying patients with CKD. Other investigators have documented state-level variations in use of uACR testing.^43^ If this resulted in geographic differences in distributions of time to CKD diagnosis, this could serve as a source of generating hypotheses about practices that lead to earlier documentation. Sixth, the diagnosis of CKD may have appeared in the free text fields of the EHR (*e.g*., problem lists or nursing notes), leading to outcome misclassification. Although it is not clear whether this misclassification would lead to bias in the time to documentation, this may point to a coding issue rather than underdiagnosis. Although this is among the first studies to account for the temporal nature of CKD diagnosis, other investigators have reported low diagnostic accuracy in patients with CKD.^22,38,44^ These estimates are not directly comparable because of differences in study design, duration of follow-up, and the operational definition of CKD. Not all quality measures include chronicity, and even among those that do, time to documentation from laboratory confirmation diagnosis is not included.^45,46^ The outcome here was similar to one of 36 peer-review performance indicators for CKD established through a Delphi process that does not include measuring chronicity: percentage of patient with a GFR value <60 ml/min per 1.73 m^2^ for 3 months, diagnosed with CKD.^47^ Delays may reflect gaps in continuity of care^48^ or failures in communication^37^ because of alert fatigue.^49^

The delays in documentation observed in this study add to the evidence of the suboptimal care patterns among patients with CKD seen in primary care.^50^ Even after CKD diagnosis, there are large proportions of patients who do not receive guideline-recommended therapies.^51^ Use of renin‐angiotensin‐aldosterone system inhibitors and statins among patients with CKD increased at diagnosis but remained <50% in between 2010 and 2020.^52^ Assessing the uptake of those recommendations and practice points would build on the evidence provided here to provide new insights into improving care in CKD. In light of emerging treatment options in CKD, optimizing care using evidence-based guideline-recommended therapies offers the opportunity of reducing the risk of cardiorenal syndrome. Future studies are needed to understand the implications of delay in documented diagnosis on medication initiation among patients with CKD.

## Supplementary Material

SUPPLEMENTARY MATERIAL

## Data Availability

Data belong to a third party, and authors are not authorized to share the data. Third Party: Optum Laboratories. Reason for Restriction: Commercial data.

## Declarative Statements

This study is exempt from the Institutional Review Board or Ethics Committee approval, and I have provided the reason for exemption. Only de-identified, Health Insurance Portability and Accountability Act Compliant data were used, informed consent was not needed and IRB approval was not required.

## Supplemental Material

Supplemental Figure 1. Flow diagram with attrition of potentially eligible patients, reasons for exclusion, and number of included patients used to estimate time to CKD diagnosis in Optum Market Clarity 2009–2020.
