# Supplementary material for: Quantifying Time to Diagnosis of CKD in the United States: Analysis of Electronic Health Records-Linked Retrospective Claims Data
Source: Kidney360. 2025 Dec 5;7(3):560–7. doi: 10.34067/KID.0000001056 (PMC13065184; doi:10.34067/KID.0000001056)
Supplement: SUPPLEMENTARY MATERIAL [file kidney360-7-560-s001.pdf]

Supplemental Figure 1. Flow diagram with attrition of potentially eligible subjects, reasons for exclusion, and number of included subjects used to estimate time to CKD diagnosis in Optum Market Clarity 2009-2020.

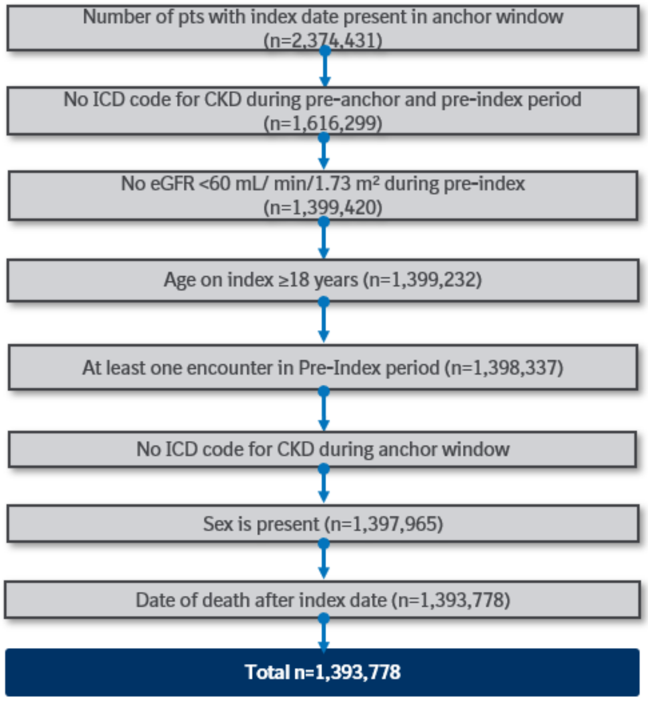

Anchor date: First eGFR <60 mL/min/1.73 m^2; Pre-anchor period: 12-month lookback period from anchor date; Anchor window: 3 -12 months from anchor date; Index date: Second eGFR <60 mL/min/1.73 m^2; Pre-index period: 12-month lookback period from index date
