# Supplementary material for: Quantifying Time to Diagnosis of CKD in the United States: Analysis of Electronic Health Records-Linked Retrospective Claims Data
Source: Kidney360. 2025 Dec 5;7(3):560–7. doi: 10.34067/KID.0000001056 (PMC13065184; doi:10.34067/KID.0000001056)
Supplement: SUPPLEMENTARY MATERIAL [file kidney360-7-560-s002.pdf]

ASN American Society of Nephrology · JASN Journal of the American Society of Nephrology · KIDNEY360 Accessing Our World From Every Angle · CJASN Clinical Journal of the American Society of Nephrology

ASN Journal Disclosure Form

As per ASN journal policy, I have disclosed any financial relationships or commitments I have held in the past 36 months as included below. I have listed my Current Employer below to indicate there is a relationship requiring disclosure. If no relationship exists, my Current Employer is not listed.

B. Donato reports the following:
Employer: Boehringer Ingelheim; and Ownership Interest: Astra Zeneca PLC.

I understand that the information above will be published within the journal article, if accepted, and that failure to comply and/or to accurately and completely report the potential financial conflicts of interest could lead to the following: 1) Prior to publication, article rejection, or 2) Post-publication, sanctions ranging from, but not limited to, issuing a correction, reporting the inaccurate information to the authors' institution, banning authors from submitting work to ASN journals for varying lengths of time, and/or retraction of the published work.

Name: Bonnie M.k. Donato
Manuscript ID: K360-2025-000558R2
Manuscript Title: Quantifying Time to Diagnosis of Chronic Kidney Disease in the United States
Date of Completion: November 3, 2025
Disclosure Updated Date: October 14, 2025

ASN American Society of Nephrology | JASN Journal of the American Society of Nephrology | KIDNEY360 Accessing Our World From Every Angle | CJASN Clinical Journal of the American Society of Nephrology

ASN Journal Disclosure Form

As per ASN journal policy, I have disclosed any financial relationships or commitments I have held in the past 36 months as included below. I have listed my Current Employer below to indicate there is a relationship requiring disclosure. If no relationship exists, my Current Employer is not listed.

C. Kovesdy reports the following:
Employer: Memphis VA Medical Center, University of Tennessee Health Science Center; Consultancy: Abbott, Ardelyx, Astra Zeneca, Bayer, Boehringer Ingelheim, Cara Therapeutics, CSL Behring, GSK, ProKidney, Renibus, Takeda; Ownership Interest: Beamlink; Research Funding: Astra-Zeneca, Bayer, Novartis; Honoraria: Abbott, Ardelyx, Astra Zeneca, Bayer, Boehringer Ingelheim, Cara Therapeutics, CSL Behring, GSK, ProKidney, Renibus, Takeda; Patents or Royalties: Royalties from UpToDate, Springer; and Advisory or Leadership Role: Associate editor: Diabetes Care; Editorial board: Kidney Medicine, Kidney International Reports; President Elect: International Society of Renal Nutrition and Metabolism.

I understand that the information above will be published within the journal article, if accepted, and that failure to comply and/or to accurately and completely report the potential financial conflicts of interest could lead to the following: 1) Prior to publication, article rejection, or 2) Post-publication, sanctions ranging from, but not limited to, issuing a correction, reporting the inaccurate information to the authors' institution, banning authors from submitting work to ASN journals for varying lengths of time, and/or retraction of the published work.

Name: Csaba P. Kovesdy
Manuscript ID: K360-2025-000558R1
Manuscript Title: Quantifying Time to Diagnosis of Chronic Kidney Disease in the United States
Date of Completion: August 21, 2025
Disclosure Updated Date: July 30, 2025

ASN American Society of Nephrology | JASN Journal of the American Society of Nephrology | KIDNEY360 Accessing Our World From Every Angle | CJASN Clinical Journal of the American Society of Nephrology

ASN Journal Disclosure Form

As per ASN journal policy, I have disclosed any financial relationships or commitments I have held in the past 36 months as included below. I have listed my Current Employer below to indicate there is a relationship requiring disclosure. If no relationship exists, my Current Employer is not listed.

A. Levy reports the following:
Employer: Dalhousie University; Consultancy: Boehringer Ingelheim; and Ownership Interest: Pelican Research Inc.

I understand that the information above will be published within the journal article, if accepted, and that failure to comply and/or to accurately and completely report the potential financial conflicts of interest could lead to the following: 1) Prior to publication, article rejection, or 2) Post-publication, sanctions ranging from, but not limited to, issuing a correction, reporting the inaccurate information to the authors' institution, banning authors from submitting work to ASN journals for varying lengths of time, and/or retraction of the published work.

Name: Adrian R. Levy
Manuscript ID: K360-2025-000558R1
Manuscript Title: Quantifying Time to Diagnosis of Chronic Kidney Disease in the United States
Date of Completion: August 21, 2025
Disclosure Updated Date: August 21, 2025

ASN American Society of Nephrology
JASN Journal of the American Society of Nephrology
KIDNEY360 Accessing Our World From Every Angle
CJASN Clinical Journal of the American Society of Nephrology

ASN Journal Disclosure Form

As per ASN journal policy, I have disclosed any financial relationships or commitments I have held in the past 36 months as included below. I have listed my Current Employer below to indicate there is a relationship requiring disclosure. If no relationship exists, my Current Employer is not listed.

S. Chatterjee reports the following:
Employer: Satabdi Chatterjee is an employee of Boehringer-Ingelheim Pharmaceuticals, Inc.; Research Funding: This research is sponsored by my current employer, Boehringer-Ingelheim.; and Other Interests or Relationships: Satabdi Chatterjee (self) - Adjunct Faculty appointment at the University of Houston College of Pharmacy, Department of Pharmaceutical Outcomes and Policy. This is an unpaid position, and involves serving as committee member in studuents' thesis and / or dissertation committees.

I understand that the information above will be published within the journal article, if accepted, and that failure to comply and/or to accurately and completely report the potential financial conflicts of interest could lead to the following: 1) Prior to publication, article rejection, or 2) Post-publication, sanctions ranging from, but not limited to, issuing a correction, reporting the inaccurate information to the authors' institution, banning authors from submitting work to ASN journals for varying lengths of time, and/or retraction of the published work.

Name: Satabdi Chatterjee
Manuscript ID: K360-2025-000558R2
Manuscript Title: Quantifying Time to Diagnosis of Chronic Kidney Disease in the United States
Date of Completion: November 3, 2025
Disclosure Updated Date: September 3, 2025

ASN
American Society of Nephrology
JASN
Journal of the American Society of Nephrology
KIDNEY360
Accessing Our World From Every Angle
CJASN
Clinical Journal of the American Society of Nephrology

ASN Journal Disclosure Form

As per ASN journal policy, I have disclosed any financial relationships or commitments I have held in the past 36 months as included below. I have listed my Current Employer below to indicate there is a relationship requiring disclosure. If no relationship exists, my Current Employer is not listed.

S. Stackland reports the following:
Employer: Panalgo; Forsyth Health

I understand that the information above will be published within the journal article, if accepted, and that failure to comply and/or to accurately and completely report the potential financial conflicts of interest could lead to the following: 1) Prior to publication, article rejection, or 2) Post-publication, sanctions ranging from, but not limited to, issuing a correction, reporting the inaccurate information to the authors' institution, banning authors from submitting work to ASN journals for varying lengths of time, and/or retraction of the published work.

Name: Sydnie Stackland
Manuscript ID: K360-2025-000558R2
Manuscript Title: Quantifying Time to Diagnosis of Chronic Kidney Disease in the United States
Date of Completion: November 21, 2025
Disclosure Updated Date: November 21, 2025

ASN American Society of Nephrology
JASN Journal of the American Society of Nephrology
KIDNEY360 Accessing Our World From Every Angle
CJASN Clinical Journal of the American Society of Nephrology

ASN Journal Disclosure Form

As per ASN journal policy, I have disclosed any financial relationships or commitments I have held in the past 36 months as included below. I have listed my Current Employer below to indicate there is a relationship requiring disclosure. If no relationship exists, my Current Employer is not listed.

L. Zhang reports the following:
Employer: Boehringer-Ingelheim

I understand that the information above will be published within the journal article, if accepted, and that failure to comply and/or to accurately and completely report the potential financial conflicts of interest could lead to the following: 1) Prior to publication, article rejection, or 2) Post-publication, sanctions ranging from, but not limited to, issuing a correction, reporting the inaccurate information to the authors' institution, banning authors from submitting work to ASN journals for varying lengths of time, and/or retraction of the published work.

Name: Ling Zhang
Manuscript ID: K360-2025-000558R2
Manuscript Title: Quantifying Time to Diagnosis of Chronic Kidney Disease in the United States
Date of Completion: November 14, 2025
Disclosure Updated Date: November 14, 2025
